# Primary Ewing's Sarcoma of the Kidney in a 73-Year-Old Man

**DOI:** 10.1155/2011/978319

**Published:** 2011-06-07

**Authors:** T. B. Wedde, I. V. K. Lobmaier, B. Brennhovd, F. Lohne, K. S. Hall

**Affiliations:** ^1^Department of Oncology, The Norwegian Radium Hospital, Oslo University Hospital, P.O. Box 4953 Nydalen, 0424 Oslo, Norway; ^2^Department of Pathology, The Norwegian Radium Hospital, Oslo University Hospital, P.O. Box 4953 Nydalen, 0424 Oslo, Norway; ^3^Department of Surgery, The Norwegian Radium Hospital, Oslo University Hospital, P.O. Box 4953 Nydalen, 0424 Oslo, Norway; ^4^Department of Radiology, The Norwegian Radium Hospital, Oslo University Hospital, P.O. Box 4953 Nydalen, 0424 Oslo, Norway

## Abstract

*Objective*. Ewing's sarcoma of the kidney is rare and is usually found in young adults. We present here a single case study of Ewing's sarcoma found in an elderly man. *Material and methods*. A 73-year-old man underwent routine surgery for hydrocoele of the testis. He developed urinary obstruction symptoms, and radiological examinations revealed a tumour in the right kidney. *Results.* Microscopical, immunohistochemical, and molecular pathological analysis of the tumour was consistent with Ewing's sarcoma. FISH showed rearrangement of chromosomes 22q12 (EWSR1). The patient subsequently underwent nephrectomy followed by 6 adjuvant chemotherapy cycles. Follow-up after 7 months shows no recurrence. *Conclusion*. This case report presents not only the rare finding of Ewing's sarcoma in the kidney, but also the occurrence of this tumour entity in an elderly patient. Treatment options for the different types of renal tumours are vastly different and the need for a correct diagnosis is, therefore, vital.

## 1. Introduction

Ewing's sarcomas are high-grade malignant tumours typically found in children and adolescents and are uncommon after the age of thirty. These tumours belong to the family of small-round cell tumours and are of neuroectodermal origin. They are mostly localized in bone or soft tissue of the extremities, the pelvis and central axis, and are rarely found in visceral organs.

Ewing's sarcomas of the kidneys are rare although the incidence is increasing [1]. In this case report, we present an elderly patient with Ewing's sarcoma of the kidney. 

## 2. Material and Methods

### 2.1. Medical History

**Figure 1:**
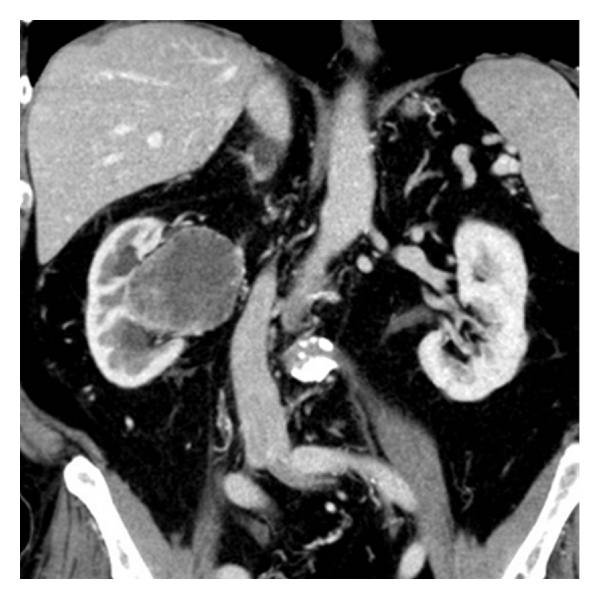
Contrast-enhanced CT abdomen and pelvis showing a tumour with central necrosis originating from the right renal pelvis and protruding into perirenal fat medially. There is slight dilatation of the superior and inferior calyces.

A 73-year-old man underwent surgery for hydrocoele of the testis. He had no other significant medical history. Due to postoperative symptoms of urinary obstruction, a CT scan was performed revealing a contrast filling tumour in the right renal pelvis and perirenal fatty tissue (Figure 1). There were no metastases present on CT imaging of thorax, abdomen, and pelvis. Fine needle biopsy with immunohistological analysis showed malignant tumour of small, round cells consistent with Ewing's sarcoma. He subsequently underwent right sided nephrectomy. Postoperative recovery was uneventful. The patient received adjuvant chemotherapy according to a modified ISG/SSG (Italian Sarcoma Group/Scandinavian Sarcoma Group). Ewing protocol treatment regimen [2] due to his age and slightly decreased kidney function following nephrectomy (Table 1). 

However, an individual assessment should always be done striving to give the same treatment regardless of the patient's age. Our patient received a total of six chemotherapy cycles. He experienced fatigue from the 6-month-long treatment regimen and did not wish to continue. His 7-month-response evaluations with CT scan and chest X-ray showed no sign of recurrence.

## 3. Results

### 3.1. Pathological Findings

#### 3.1.1. Gross Examination

Gross examination showed a large tumour in the right kidney extending into the renal pelvis and through the renal capsule into perirenal fat, but not through the Gerota's fascie. 

#### 3.1.2. Microscopic Examination

**Figure 2:**
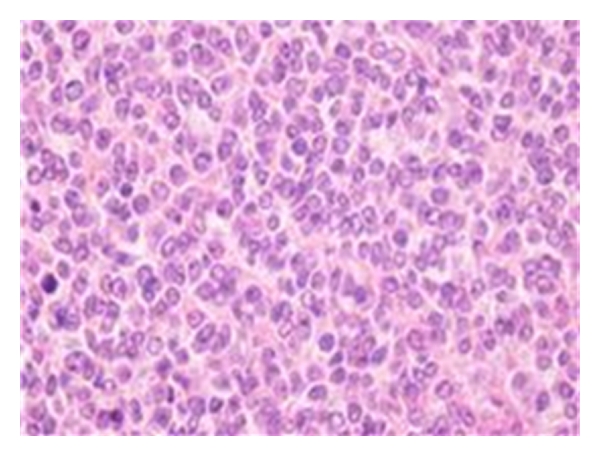
Typical histological specimen which shows sheets of small round uniform cells without clear cell boundaries and round to oval nuclei with finely dispersed chromatin and one-to-two small nucleoli (HE).

The tumour consisted of solidly packed, strikingly uniform small round cells with scanty, pale cytoplasm and round to oval nuclei with sharp borders and one to two small nucleoli (Figure 2).

#### 3.1.3. Immunohistochemical Analysis

**Figure 3:**
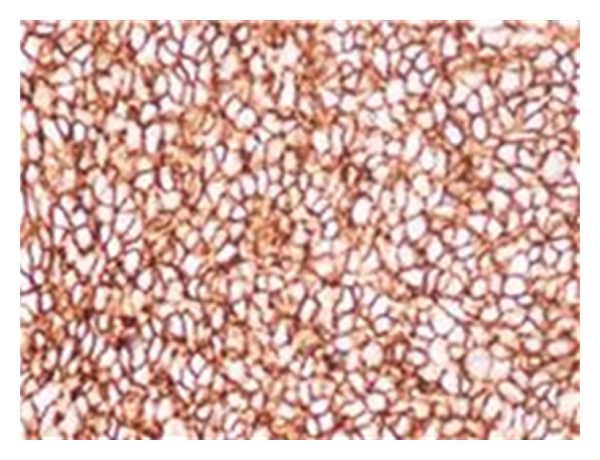
Immunohistochemical analysis shows uniform, strong positive staining for CD99.

Immunohistochemical analysis showed positive staining for vimentin, CD99 and CD 117 (Figure 3). The tumour cells were negative for WT-1, Fli-1, AE1/AE3, MYF-4, desmin, synaptophysin, chromogranin, S-100, CD56, CD3, TdT, and CD20.

#### 3.1.4. Molecular Pathological Findings

FISH showed rearrangement of chromosomes 22q12 (EWSR1). Real-time RT-PCR showed EWSR-1-FLI1 or EWSR1-ERG genfusion consistent with Ewing's sarcoma, supporting the FISH findings.

## 4. Discussion

Ewing's sarcoma of the kidney is rare. The majority are seen in young adults with a mean age of presentation between 28 and 34 years (range 4–69 years), and a slight male predominance [3]. In Norway, there are 5–10 new reported cases of Ewing's sarcomas (all locations) annually (Norwegian Cancer Registry).

Ewing's sarcoma (or Primitive Neuro Ectodermal Tumour, PNET) belongs to a family of small round-cell tumours known as “The Ewing family of tumours.” They are derived from neuroectodermal cells and are localized both in soft tissue, visceral organs, and bone, the latter more commonly. PNET's can occur in numerous visceral organs including urogenital, intra-abdominal and intrathoracic organs, with kidney being the most common [4, 5]. 

Renal cell carcinoma is the most common renal tumour and accounts for approximately 85% of all renal tumours and 2% of all new cancer cases in Norway according to data from the Norwegian Cancer Registry; hence, renal cell carcinoma has to be ruled out when finding a renal tumour in an elderly patient (>50 years of age). If the tumour shows a small round-cell pattern as in our case, the differential diagnosis ranges from malignant lymphomas, small cell carcinoma, small cell osteosarcoma, rhabdomyosarcoma, synovial sarcoma, and desmoplastic small round-cell tumours. 

When diagnosing Ewing's sarcoma, the combination of morphological findings, immunohistochemical analyses, and genetic changes together forms the base of the diagnosis. Our patient had a tumour that consisted of small round cells which stained positive for CD99. FISH showed the classic rearrangement that is seen in Ewing's sarcoma, and PCR confirmed this finding.

Ewing's sarcoma/PNET has diagnostic genetical findings. The most common translocation is t(11; 22) (q24; q12) with EWSR1-FLI1 genfusion (>90%) [3, 6, 7]. EWSR1 rearrangement can occur in other malignant tumours. In our case, the most relevant differential diagnosis is the very rare desmoplastic small round-cell tumour which can look much like Ewing's sarcoma morphologically. When FISH shows this rearrangement, PCR can help determine which tumour it is when the morphological findings are similar. 

There are different views of the value of adjuvant chemotherapy in this group of patients. Several studies show the benefit of adjuvant chemotherapy [8–10] whilst others find no clinical improvement [3]. Our patient was given six cycles of chemotherapy, but it is still too early to determine the outcome of his treatment.

 Ewing's sarcoma/PNET of the kidney is an aggressive tumour, and 5-year disease-free survival is reported around 45–55% [11–15]. Mukkunda et al. [16] found in their analysis of 7 patients with renal Ewing's sarcoma and with median follow-up of 36 months (range from 5 to 149), a median disease-free survival in patients with nonmetastatic disease of 30.35 months (range from 5.1 to 149) with a 5-year overall survival rate of 42%. All PNETs show a 5-year survival rate of 58–61% with a median survival of 120 months [5]. In comparison, Gupta et al. [17] found in their analysis of adult patients with localised Ewing's sarcoma in bone and soft tissue a 3-year event-free survival of 43% +/− 13%. Further follow-up of these patients is required to rule out relapse of the tumour as Ewing's sarcomas are highly aggressive tumours.

## 5. Conclusion

Ewing's sarcoma of the kidney is in itself a rare diagnosis. This tumour is generally seen in young adults in bone and soft tissue of the extremities and pelvis. This case shows that Ewing's sarcomas in the kidney may occur in all ages. 

The treatment options for the different types of renal tumours are vastly different and the need for a correct diagnosis is, therefore, vital. The best practice treatment today is based on both surgical dissection and chemotherapy.

##  Conflict of Interests

The authors declared that there is no conflict of interests.

## Figures and Tables

**Table 1:** Total dose of chemotherapy drugs per square meter.

	mg/m^2^
Vincristine	3. 1
Etoposide	302
Actinomycin D	1,04
Cyclofosfamide	1677
Doxorubicin	265
Ifosfamide	12369
